# The psychology behind physics teachers’ attitudes toward out-of-school learning: the roles of self-efficacy and anxiety

**DOI:** 10.3389/fpsyg.2026.1951607

**Published:** 2026-10-22

**Authors:** Tolga Saka

**Affiliations:** Department of Physics Education, Dede Korkut Faculty of Education, Kafkas University, Kars, Türkiye

**Keywords:** anxiety, attitude, out-of-school learning, physics teachers, self-efficacy

## Abstract

**Introduction:**

This study aimed to examine physics teachers’ attitudes toward out-of-school learning, self-efficacy beliefs, and anxiety in terms of various demographic variables, and to determine whether their self-efficacy and anxiety regarding out-of-school learning are significant predictors of their attitudes toward out-of-school learning.

**Methods:**

An explanatory sequential mixed-methods research design was employed. Quantitative data were collected from 41 physics teachers employed by the Ministry of National Education in a province of Türkiye using the Self-Efficacy Belief Scale for Organizing Educational Field Trips to Out-of-School Learning Environments, the Anxiety Scale, and the Attitude Scale, all of which were developed by experts in the field. Following the quantitative phase, semi-structured interviews were conducted with 16 teachers selected from the quantitative sample through maximum variation sampling to explore their views and experiences in greater depth. Quantitative data were analyzed using descriptive statistics, independent samples *t*-tests, correlation analysis, and multiple linear regression analysis, whereas qualitative data were analyzed using content analysis.

**Results:**

A statistically significant gender difference was found in attitudes toward out-of-school learning, with male teachers reporting higher scores than female teachers. The remaining demographic comparisons were not statistically significant; however, given the limited sensitivity associated with the small subgroup sizes, these results were considered inconclusive rather than evidence of no group differences. Self-efficacy was identified as a significant positive predictor of attitudes toward out-of-school learning, whereas anxiety was found to be a significant negative predictor. Together, self-efficacy and anxiety accounted for 39% of the variance in attitude scores in this sample (*R*^2^ = 0.39; adjusted *R*^2^ = 0.36). The qualitative findings further indicated that teachers’ anxiety was primarily associated with student safety, legal procedures, administrative responsibilities, curriculum intensity, limited resources, and a lack of practical implementation experience.

**Discussion:**

In this sample, self-efficacy and anxiety were associated with attitudes toward out-of-school learning, while the demographic comparisons identified a significant gender difference in attitude scores but no other statistically significant group differences. Whether support aimed at teachers’ self-efficacy and implementation-related concerns increases the use of out-of-school learning requires further investigation.

## 1 Introduction

In recent years, the view that classroom instruction alone is insufficient to foster students’ scientific thinking, problem-solving, real-life application, and meaningful learning has gained increasing prominence. In particular, the decline in students’ interest, motivation, and attitudes toward science and physics courses as they progress through educational levels has increased interest in alternative learning environments (Bennett, 2003). In this context, out-of-school learning environments are regarded as important educational settings where students can experience scientific concepts in authentic real-life contexts, actively participate in the learning process, and assume greater responsibility for their own learning (Beames et al., 2012). Out-of-school learning environments support students not only in acquiring knowledge but also in observing, investigating, exploring, and developing their scientific process skills (Hein, 2009). The educational psychology literature suggests that learning environments in which students can actively participate, interact with their surroundings, and engage in authentic real-life experiences promote higher levels of intrinsic motivation (Ryan and Deci, 2020). According to Self-Determination Theory, learning environments that support individuals’ needs for autonomy, relatedness, and competence enhance motivation for learning. In this respect, out-of-school learning environments contribute to satisfying students’ basic psychological needs by enabling them to take more active roles in the learning process, increasing their experiences of autonomy, and encouraging active participation (Ryan and Deci, 2000; Salmi and Thuneberg, 2019). Research has demonstrated that out-of-school learning activities positively influence students’ attitudes toward courses, motivation, and academic achievement (Fägerstam and Grothérus, 2018). Similarly, Renninger and Hidi (2015) argued that these learning experiences contribute to sustaining students’ attention, increasing the value they attribute to learning, and promoting voluntary information-seeking behaviors.

Physics instruction involves abstract thinking, conceptual reasoning, problem-solving, and scientific reasoning processes to a considerable extent (Gaigher et al., 2007). Therefore, it is particularly important for students to establish connections between real-life phenomena and the physics concepts they learn in the classroom. Ertas et al. (2011) reported that out-of-school instructional activities on the topic of energy enhanced students’ understanding of energy concepts and their ability to relate these concepts to everyday life. Furthermore, out-of-school learning activities have been reported to exert positive effects on students’ scientific process skills, inquiry skills, and problem-solving competencies (Ainsworth and Eaton, 2010).

The positive effects of out-of-school learning environments on students largely depend on teachers’ competence in planning, organizing, and implementing these learning environments. Therefore, one of the fundamental factors determining the success of out-of-school learning practices is teachers’ psychological characteristics related to this process. Teachers’ attitudes toward out-of-school learning, self-efficacy beliefs, and anxiety constitute important psychological variables influencing the feasibility and implementation of such instructional processes. The educational psychology literature emphasizes that teachers’ instructional behaviors are shaped not only by their pedagogical knowledge but also by their self-efficacy beliefs, motivation, anxiety, and psychological readiness (Bandura, 1997; Zee and Koomen, 2016). In this context, physics teachers’ self-efficacy perceptions regarding the planning, organization, and implementation of out-of-school learning activities may be relevant to their willingness to undertake such activities.

Teachers’ attitudes toward out-of-school learning are relevant to whether they use these environments for instruction, while careful planning and alignment with learning objectives are important considerations when organizing such activities (Henriksson, 2018; Rickinson et al., 2004). Conversely, when teachers develop negative attitudes toward out-of-school learning or perceive such practices as a waste of time, the implementation of out-of-school learning activities may be constrained (Fägerstam, 2014; Oberle et al., 2021). In addition, teachers’ anxiety toward out-of-school learning is regarded as a noteworthy psychological variable. Teachers may experience anxiety about implementing out-of-school learning activities due to factors such as student safety, legal procedures, bureaucratic processes, time pressure, classroom management, and parental expectations (Yıldırım and Efendıoglu, 2023). From the perspective of educational psychology, anxiety is considered an emotional response arising from uncertainty, perceived loss of control, and a high sense of responsibility (Lazarus and Folkman, 1984). In a study of classroom teachers, interview participants reported avoiding out-of-school trips because of concerns about potential harm and student safety, despite recognizing the benefits of these activities (Yıldırım and Efendıoglu, 2023). Taken together, these findings suggest that teachers’ attitudes toward out-of-school learning are associated not only with pedagogical knowledge but also with psychological processes such as self-efficacy and anxiety.

In this study, attitudes toward out-of-school learning were treated as the outcome variable, reflecting teachers’ overall evaluations of this instructional approach. This modeling choice was grounded in Social Cognitive Theory and the teacher self-efficacy literature, which suggest that teachers’ perceived capabilities and implementation-related emotional experiences may be associated with their evaluations of instructional practices (Bandura, 1997; Tschannen-Moran and Hoy, 2001; Zee and Koomen, 2016). Within this framework, self-efficacy represents teachers’ perceived capability to plan and conduct out-of-school learning activities, whereas anxiety represents their emotional evaluations of the potential difficulties and risks associated with implementing these activities. Accordingly, the current regression model was specified to examine how self-efficacy and anxiety are associated with teachers’ attitudes toward out-of-school learning. However, the theoretical relationship among these variables is not assumed to be unidirectional. According to Control-Value Theory, anxiety may arise when individuals perceive insufficient control over an activity that they consider important or valuable (Pekrun, 2006). From this perspective, the value teachers attribute to out-of-school learning and their evaluations of this instructional approach may also be associated with their anxiety. Therefore, a reverse modeling direction in which attitude is considered an antecedent of anxiety is also theoretically defensible. The direction specified in the present study represents an analytical and theoretical modeling choice aligned with the research question; the cross-sectional research design does not demonstrate that self-efficacy or anxiety precedes attitudes or causes changes in attitudes.

Teachers’ gender, professional experience, type of faculty graduated from, and workplace location may influence their perceptions of instructional processes and their psychological dispositions. In particular, teachers working in rural areas may encounter different environmental opportunities and experiences (Vijayavarathan-R et al., 2022), more experienced teachers may possess greater practical implementation experience (Tschannen-Moran and Hoy, 2001), and teachers graduating from different types of faculties may develop different perceptions of competence in terms of pedagogical or subject matter knowledge (Bümen and Özaydın, 2013). Therefore, it is important to examine teachers’ psychological characteristics related to out-of-school learning in terms of demographic variables.

A review of the literature on out-of-school learning indicates that previous studies have predominantly focused on science teachers, preservice teachers, or research conducted within the broader context of science education (Henriksson, 2018). Previous studies have examined relevant psychological constructs in other teacher groups: Yıldırım and Efendıoglu (2023) investigated the relationship between self-efficacy and anxiety among classroom teachers, while Arık (2022) and Utku and Uçak (2023) examined anxiety related to out-of-school learning among science teachers. Building on this research, the present study examines the associations of self-efficacy and anxiety with attitudes toward out-of-school learning specifically among physics teachers. Examining teachers’ decisions regarding out-of-school learning not only in terms of demographic characteristics but also in conjunction with psychological processes such as self-efficacy and anxiety may provide a more comprehensive perspective for promoting the implementation of these instructional practices. Therefore, there is a need for comprehensive research that integrates these psychological constructs influencing teachers’ implementation of out-of-school learning within a single conceptual framework. In this context, this study aimed to examine physics teachers’ attitudes toward out-of-school learning, their self-efficacy beliefs regarding the organization of out-of-school learning activities, and their anxiety in terms of various demographic variables. In addition, the study investigated whether teachers’ self-efficacy and anxiety toward out-of-school learning are significant predictors of their attitudes toward out-of-school learning. Accordingly, by addressing physics teachers’ psychological characteristics related to out-of-school learning from a holistic perspective, the study aims to contribute to both the out-of-school learning literature and the field of educational psychology.

### 1.1 Research question

Do physics teachers’ attitudes toward out-of-school learning, self-efficacy beliefs, and anxiety differ according to various demographic variables, and do these psychological variables significantly predict teachers’ attitudes toward out-of-school learning?

To address this research question, the following sub-research questions were investigated:

- Do physics teachers’ attitudes toward out-of-school learning activities differ according to gender, professional experience, type of faculty graduated from, and workplace location?
- Do physics teachers’ self-efficacy beliefs regarding their ability to organize educational field trips to out-of-school learning environments differ according to gender, professional experience, type of faculty graduated from, and workplace location?
- Do physics teachers’ anxiety regarding organizing field trips to out-of-school learning environments differ according to gender, professional experience, type of faculty graduated from, and workplace location?
- Are physics teachers’ self-efficacy and anxiety regarding out-of-school learning significant predictors of their attitudes toward out-of-school learning?

## 2 Method

### 2.1 Research design

An explanatory sequential mixed-methods design was used, with quantitative data collection and analysis followed by semi-structured interviews. This research approach involves collecting and analyzing quantitative and qualitative data within the same study and integrating the findings (Creswell and Plano Clark, 2018). In the first phase, quantitative survey data were collected to examine demographic group differences and the relationships among teachers’ attitudes toward out-of-school learning, self-efficacy, and anxiety. In the second phase, semi-structured interviews were conducted with teachers selected from the quantitative sample through maximum variation sampling based on their demographic characteristics. Participants for the interview phase were selected on the basis of demographic variation rather than their individual scale scores. The interviews were used to provide contextual insight into the attitudes, perceived capabilities, and concerns measured in the quantitative phase. The quantitative and qualitative components were integrated during the reporting and interpretation of the findings: for each of the first three research questions, statistical group comparisons were followed by interview themes and participant quotations. Relevant interview themes were also considered when discussing the relationships identified in the correlation and regression analyses.

### 2.2 Sample

The sample of the study consisted of 41 physics teachers employed by the Ministry of National Education in a province of Türkiye. The study sample was selected from the target population based on voluntary participation using convenience and purposive sampling methods. Purposive sampling is a method used to identify information-rich cases that enable an in-depth investigation of the phenomenon within the context of the research objectives. Convenience sampling, on the other hand, involves selecting participants from units that are readily accessible and feasible to implement due to limitations related to cost, workforce, or time in the research process (Büyüköztürk et al., 2018). The demographic characteristics of the participating teachers are presented in Table 1.

**Table 1:** Demographic characteristics of the participating teachers.

Variable	Group	*f*	%
Gender	Female	14	34.1
	Male	27	65.9
Teaching experience	14 years or less	19	46.3
	15 years or more	22	53.7
Faculty of graduation	Faculty of education	16	39.0
	Faculty of arts and sciences	25	61.0
School location	City center	22	53.7
	Rural area	19	46.3
Total		41	100

According to Table 1, approximately two-thirds of the participants were male teachers. The teachers who participated in the study were divided into two groups with the closest number of participants in terms of years of professional experience. In addition, only approximately two-fifths of the participants were graduates of faculties of education, and more than half were employed in the city center.

For the qualitative phase, 16 teachers were selected from the 41 participants in the quantitative phase using maximum variation sampling. Four characteristics were considered: gender (female or male), teaching experience (14 years or less or 15 years or more), faculty of graduation (faculty of education or faculty of arts and sciences), and school location (city center or rural area). The combinations of these characteristics yielded 16 possible profiles, and one teacher representing each profile participated in a semi-structured interview. The demographic profiles of the 16 interview participants are presented in Table 2.

**Table 2:** Sample group representing maximum diversity.

Participant code	Gender	Faculty of graduation	School location	Teaching experience
P1	Female	Faculty of education	Rural area	14 years or less
P2	Male	Faculty of education	City center	14 years or less
P3	Female	Faculty of education	City center	15 years or more
P4	Male	Faculty of education	Rural area	14 years or less
P5	Female	Faculty of arts and sciences	City center	14 years or less
P6	Male	Faculty of arts and sciences	City center	14 years or less
P7	Female	Faculty of arts and sciences	Rural area	15 years or more
P8	Male	Faculty of education	Rural area	15 years or more
P9	Male	Faculty of arts and sciences	Rural area	15 years or more
P10	Female	Faculty of arts and sciences	City center	15 years or more
P11	Female	Faculty of education	City center	14 years or less
P12	Male	Faculty of arts and sciences	City center	15 years or more
P13	Female	Faculty of education	Rural area	15 years or more
P14	Male	Faculty of arts and sciences	Rural area	14 years or less
P15	Female	Faculty of arts and sciences	Rural area	14 years or less
P16	Male	Faculty of education	City center	15 years or more

### 2.3 Data collection instruments

Both quantitative and qualitative data collection instruments were employed to achieve the purpose of the study. Among the quantitative data collection instruments, the “Self-Efficacy Belief Scale for Organizing Educational Field Trips to Out-of-School Learning Environments” consists of 30 items, and the Cronbach’s Alpha reliability coefficient of the scale was calculated as 0.93 (Bozdoğan, 2016). The “Teacher Anxiety Scale for Organizing Field Trips to Out-of-School Learning Environments”, developed by Arık and Bozdoğan (2022), consists of 28 items, and its Cronbach’s Alpha reliability coefficient was determined to be 0.944 (Arık and Bozdoğan, 2022). In addition, the “Teacher Attitude Scale toward Out-of-School Learning Activities”, developed by Göloğlu Demir and Çetin (2021), consists of 25 items, and the overall Cronbach’s Alpha reliability coefficient of the scale was found to be 0.92. For the present sample, Cronbach’s alpha was 0.86 for the 30-item self-efficacy scale (*n* = 41), 0.87 for the 28-item anxiety scale (*n* = 41), and 0.89 for the 25-item attitude scale (*n* = 41).

As the qualitative data collection instrument, the *semi-structured interview form* developed by the researcher was employed. At the beginning of the development process of the form, the researcher prepared a pool of questions that were considered capable of revealing the views of the relevant teachers in line with the purpose of the study. To ensure the content validity of the interview form, the opinions of three experts in physics education were obtained. Based on the experts’ feedback, an interview form consisting of 36 questions was developed. Subsequently, the forms were sent online to three additional physics education experts, different from the initial experts, and the content validity and usability of the interview form were examined. As part of these evaluations, some questions in the interview form were removed, while others were merged, reducing the total number of questions in the form to 27. The linguistic accuracy of the form was reviewed by an academic specializing in Turkish language education. Consequently, the interview form to be used to determine physics teachers’ views on out-of-school learning, in line with the purpose of the study, consisted of 27 questions.

### 2.4 Data collection process

It was considered that administering more than one scale to physics teachers at the same time might cause them to become fatigued and, consequently, not respond to the questionnaire items with the necessary level of attention. To prevent this situation, these three scales were administered to the physics teachers at three different times. The semi-structured interview form was administered after the completion of the quantitative data collection process with physics teachers selected to ensure maximum variation by taking their demographic characteristics into consideration. Administering the scales at different times also separated the measurement occasions, although all three measures remained self-reports completed by the same teachers.

The semi-structured interviews were conducted face-to-face after quantitative data collection was completed. Each interview lasted approximately 60 min. Participants’ responses were audio-recorded with their consent. The recordings were subsequently transcribed and transferred to electronic documents.

### 2.5 Data analysis

The quantitative data obtained within the scope of the study were analyzed in accordance with the research questions, and all statistical analyses were conducted using the SPSS 27.0 statistical software package. Initially, descriptive statistics (mean, standard deviation, and skewness coefficient) were calculated for the dataset. The normality of the variables was evaluated based on the skewness coefficients, and skewness values within the range of ±1 were accepted as indicating a normal or approximately normal distribution of the data (Tabachnick and Fidell, 2013).

After the assumption of normality had been satisfied, an independent samples *t*-test was used to compare the groups with respect to variables consisting of two independent groups (gender, professional experience, type of faculty graduated from, and workplace location). Homogeneity of variance was assessed using Levene’s test. The conventional independent-samples Student’s *t*-test was reported when the assumption of equal variances was met (*p* > 0.05). Welch’s *t*-test, which does not assume equal population variances, was used when Levene’s test indicated unequal variances (*p* < 0.05). Cohen’s *d* was reported as the standardized effect size for the independent-group comparisons, together with its 95% confidence interval. Given the fixed sample size and relatively small demographic subgroups, a sensitivity power analysis was conducted for the independent-group comparisons using a two-tailed test, *α* = 0.05, and 80% power. Given the observed subgroup sizes (ranging from 14 to 27 participants), the minimum detectable standardized mean difference was approximately *d* = 0.90–0.95 across the demographic comparisons. Thus, the study had limited sensitivity to detect small-to-moderate group differences, and non-significant demographic comparisons were interpreted as inconclusive rather than as evidence of equivalence between groups.

Pearson’s product–moment correlation analysis was conducted to determine the relationships among the variables included in the study. In addition, before conducting the regression analysis, the presence of a multicollinearity problem among the independent variables was examined.

Multiple linear regression analysis was performed to determine the predictive effects of the independent variables on the dependent variable. Before proceeding with the regression analysis, the required assumptions were tested. Scatterplots were inspected for patterns in the relationships between the predictors and attitude scores and in the standardized residuals plotted against standardized predicted values. A normal P–P plot of the standardized residuals was inspected to assess whether the residuals were approximately normally distributed. The regression model was then fitted, and the coefficients (*B* and *β*), *t* values, and significance levels (*p*) were reported.

The qualitative data obtained from the semi-structured interviews conducted with the group of teachers selected through maximum variation sampling were analyzed using the content analysis method. Content analysis is defined as the systematic interpretation of textual data through coding and the identification of themes or patterns (Hsieh and Shannon, 2005). During the analysis process, the interview transcripts were read repeatedly in accordance with the research questions. Subsequently, themes and codes were generated from the data, and codes with similar meanings were grouped under the relevant themes. The consistency among the themes was continuously reviewed using the constant comparative analysis approach, and the necessary revisions were made. Three faculty members with expertise in the field also analyzed the interview data. Their codes and themes were compared, and differences were discussed to establish a shared set of codes and themes. The researcher then compared their own coding with this shared coding. Following Miles and Huberman (1994), the percentage of agreement was calculated as the number of agreed code assignments divided by the total number of agreed and disagreed code assignments, multiplied by 100. The resulting agreement percentages ranged from 85% to 95%. According to Miles and Huberman (1994), agreement rates above 70% are considered indicative of an acceptable level of reliability. Accordingly, the analysis process was considered to be reliable.

## 3 Findings

The findings are organized according to the research questions. For the questions concerning demographic group differences in attitudes, self-efficacy, and anxiety, the quantitative results are followed by interview themes and participant quotations. The correlation and regression results are presented separately. In the Discussion section, the relevant quantitative and qualitative findings were explicitly considered together to identify patterns of convergence, divergence, or complementarity.

### 3.1 Findings regarding the examination of physics teachers’ attitudes toward out-of-school learning activities in terms of gender, professional experience, type of faculty graduated from, and workplace location

Physics teachers’ attitudes toward out-of-school learning activities were examined in terms of gender, professional experience, type of faculty graduated from, and workplace location. Accordingly, the findings obtained from the quantitative and qualitative data are presented using a holistic approach and following a systematic sequence.

To determine whether the participating teachers’ scores on the Teacher Attitude Scale Toward Out-of-School Learning Activities (TASOLA) differed according to gender, professional experience, type of faculty graduated from, and workplace location groups, descriptive statistics were first calculated, and the normality of the score distributions was examined. The univariate normality of the participants’ TASOLA scores according to the groups of the independent variables examined in the study was evaluated based on the skewness coefficient. The descriptive statistics calculated for the participating teachers’ TASOLA scores according to the groups of the independent variables, together with the results of the analyses, are presented in Table 3.

**Table 3:** Analysis results of the participants’ TASOLA scores according to the groups of the independent variables.

Variable	Group	*N*	*M*	*SD*	Skewness	Test	*t*	*df*	*p*	Cohen’s *d*	95% CI for *d*
Gender	Female	14	3.53	0.17	0.08	Welch	−2.56	33.45	0.02	−0.64	[−1.29, 0.03]
	Male	27	3.83	0.57	0.08						
Teaching experience	14 years or less	19	3.62	0.41	0.22	Student	−1.36	39	0.18	−0.43	[−1.05, 0.20]
	15 years or more	22	3.83	0.54	0.57						
Faculty of graduation	Faculty of education	16	3.60	0.28	0.89	Welch	−1.62	36.77	0.12	−0.45	[−1.08, 0.19]
	Faculty of arts and sciences	25	3.81	0.58	0.21						
School location	City center	22	3.69	0.48	0.35	Student	−0.62	39	0.54	−0.19	[−0.81, 0.42]
	Rural area	19	3.78	0.50	0.91						

Welch’s *t*-test was used when the assumption of homogeneity of variance was violated. The 95% confidence intervals for Cohen’s *d* were reported for all independent-group comparisons. In the Findings section, however, the confidence intervals reported alongside Welch’s test refer to the raw mean difference between the groups rather than to the standardized effect size.

The analysis results presented in Table 3 indicate that the TASOLA scores of the teachers who participated in the study exhibited a normal or approximately normal distribution across the demographic variable groups. A variable is considered to be approximately normally distributed when its skewness value is less than plus or minus one (−1 < skewness < +1) (Tabachnick and Fidell, 2013). Levene’s test indicated that the assumption of homogeneity of variance was violated for comparisons based on gender (*F* = 12.93, *p* = 0.001) and type of faculty graduated from (*F* = 7.26, *p* = 0.010). Therefore, Student’s *t*-test was used for comparisons based on professional experience and workplace location, whereas Welch’s *t*-test was used for comparisons based on gender and type of faculty graduated from. The analysis indicated a statistically significant difference in TASOLA scores according to gender, Welch’s *t* (33.45) = −2.56, *p* = 0.015, 95% CI [−0.54, −0.06]. Male teachers (*M* = 3.83, *SD* = 0.57) reported higher attitude scores than female teachers (*M* = 3.53, *SD* = 0.17). The difference in TASOLA scores according to type of faculty graduated from was not statistically significant, Welch’s *t* (36.77) = −1.62, *p* = 0.115, 95% CI [−0.49, 0.06]. The comparisons based on professional experience and workplace location were not statistically significant (*p* > 0.05); however, given the limited sensitivity of the study, these non-significant results should be regarded as inconclusive rather than as evidence of no group difference.

The findings obtained from the analysis of the qualitative data regarding physics teachers’ attitudes toward out-of-school learning activities are presented below Table 4, supported by representative excerpts from the teacher interview data.

**Table 4:** Physics teachers’ attitudes toward out-of-school learning activities according to demographic variables.

Theme	Code	Sub-codes		Illustrative participants							
				Female	Male	≤14 years	≥15 years	Faculty of education	Faculty of arts and sciences	City center	Rural area
Attitude	Belief in its usefulness	Promoting lasting learning	Relating learning to daily life	✓	✓	✓	✓	✓	✓	✓	✓
			Instruction based on concretization	✓	✓		✓		✓	✓	✓
			Experiential learning	✓	✓	✓	✓	✓	✓	✓	✓
			Moving beyond rote learning		✓		✓		✓		✓
		Enhancing motivation, curiosity, and interest		✓	✓	✓	✓	✓	✓	✓	✓
		Supporting students’ social development		✓	✓	✓	✓	✓	✓	✓	✓
		Higher-order skill development		✓	✓		✓		✓	✓	✓
		Fostering self-confidence		✓	✓		✓		✓		✓
		Fostering a sense of responsibility		✓			✓		✓		✓
		Providing opportunities for career exploration		✓	✓		✓		✓	✓	✓
	Suitability for physics instruction	Perceived suitability for physics instruction			✓		✓		✓		✓
		Perceived unsuitability for physics instruction			✓	✓		✓		✓	
	Level of interest and willingness	Student-related factors	Interest-based participation	✓	✓	✓	✓		✓	✓	✓
		Willingness to implement		✓			✓		✓	✓	✓
		Availability of favorable conditions			✓		✓		✓	✓	
		Unwillingness	Legal and administrative procedures	✓		✓		✓			✓
		Belief that it is optional		✓		✓		✓			✓

A check mark indicates that at least one interviewed teacher in the demographic group expressed the corresponding subcode. The same teacher may be represented under multiple subcodes. Check marks do not indicate participant frequencies or the prevalence of a view within a group.

When Table 4 is examined, several attitude-related views are represented in interview accounts across the demographic categories. In the “Belief in Its Usefulness” category, interview accounts from each demographic group included references to lasting learning, increased student motivation, interest and curiosity, and support for students’ social development. In particular, a view expressed across all demographic groups was that these activities enable students to connect learning with everyday life and engage in experiential learning. This finding is supported by the statement of P9, a male teacher who graduated from a faculty of arts and sciences and works in a rural area, “I think they will be successful as they experience it.”, the statement of P1, a female teacher who graduated from a faculty of education and works in a rural area, “Since students learn by doing and experiencing, learning becomes more permanent.”, the statement of P4, who has 14 years or less of professional experience, “When students learn by doing and experiencing, learning becomes more permanent.”, and the statement of P8, who has 15 years or more of professional experience, “Learning by doing is more permanent.” Some codes, however, were represented only in interview accounts from particular demographic categories. These distributions indicate the demographic categories in which particular views were represented in the interview accounts; they should not be interpreted as evidence of differences between demographic groups. Within the Promoting Lasting Learning code, it was determined that teachers with 14 years or less of professional experience and those who graduated from faculties of education did not refer to the sub-codes of Instruction Based on Concretization and Moving Beyond Rote Learning. In addition, teachers working in city centers and female teachers did not refer to the subcode of Moving Beyond Rote Learning. Furthermore, graduates of faculties of education and teachers with 14 years or less of professional experience did not refer to the codes of Higher-Order Skill Development, Fostering a Sense of Responsibility, and Providing Opportunities for Career Exploration. The Fostering Self-Confidence code was not mentioned by graduates of faculties of education, teachers with 14 years or less of professional experience, or teachers working in city centers. It was also determined that male teachers and those working in the city center did not refer to the code of Fostering a Sense of Responsibility.

Although positive views on the usefulness of out-of-school learning were represented in accounts from both teaching-experience categories, contrasting views were identified regarding its suitability for physics instruction. The view that out-of-school learning was suitable for physics instruction was expressed by P9. P9 was a male teacher with 15 or more years of professional experience, had graduated from a faculty of arts and sciences, and was working in a rural area. In contrast, the view that out-of-school learning was not suitable for physics instruction was expressed only by P2. P2 was a teacher with 14 years or less of professional experience, had graduated from a faculty of education, and was working in a city center. These contrasting views are illustrated by P9, a male teacher with at least 15 years of professional experience who graduated from a faculty of arts and sciences and works in a rural area: “Physics is already a subject closely connected with nature. It is highly suitable for out-of-school activities.” P2, a graduate of a faculty of education with 14 years or less of professional experience who works in a city center, stated: “It is generally not suitable for physics”.

In the Level of Interest and Willingness category, some teachers’ willingness to implement out-of-school learning activities was found to depend on students’ interests and needs. The statement of P9, a male teacher, “If the student is willing, I will do it as well.”, and the statement of P5, a female teacher who graduated from a faculty of arts and sciences and has 14 years or less of professional experience, “Whether I implement it depends on whether the students want it.” support the codes of Student-Related Factors and Interest-Based Participation. In contrast, P1 expressed the view that legal permission procedures were a factor limiting willingness to implement out-of-school learning activities. P1 was a female teacher with 14 years or less of professional experience, had graduated from a faculty of education, and was working in a rural area. The statement of P1, a female teacher who graduated from a faculty of education and has 14 years or less of professional experience, “The permission procedures are a very long and exhausting process. They wear you out.” P1’s account illustrates how permission procedures were perceived as a constraint on willingness to organize these activities. Other interview accounts described willingness to implement these activities as dependent on favorable conditions. P12, for example, stated: “If there is a suitable environment and the conditions are favorable, I will do it. It would be beneficial”.

### 3.2 Findings regarding the examination of physics teachers’ self-efficacy for organizing educational field trips to out-of-school learning environments in terms of gender, professional experience, type of faculty graduated from, and workplace location

Physics teachers’ self-efficacy in organizing educational trips to out-of-school learning environments was examined in relation to gender, teaching experience, type of faculty from which they graduated, and school location. Accordingly, the findings obtained from the quantitative and qualitative data are presented using a holistic approach and following a systematic sequence.

To determine whether the participating teachers’ scores on the Teacher Self-Efficacy Scale Toward Out-of-School Learning (TSESOSL) differed according to gender, professional experience, type of faculty graduated from, and workplace location groups, descriptive statistics were first calculated, and the normality of the score distributions was examined. The univariate normality of the participants’ TSESOSL scores according to the groups of the independent variables examined in the study was evaluated based on the skewness coefficient. The descriptive statistics calculated for the participating teachers’ TSESOSL scores according to the groups of the independent variables, together with the results of the analyses, are presented in Table 5.

**Table 5:** Analysis results of the participants’ TSESOSL scores according to the groups of the independent variables.

Variable	Group	*N*	*M*	*SD*	Skewness	Test	*t*	*df*	*p*	Cohen’s *d*	95% CI for *d*
Gender	Female	14	3.94	0.20	0.57	Welch	−1.27	37.08	0.21	−0.33	[−0.98, 0.32]
	Male	27	4.08	0.51	−0.41						
Teaching experience	14 years or less	19	3.94	0.37	−0.72	Student	−1.29	39	0.20	−0.41	[−1.02, 0.22]
	15 years or more	22	4.11	0.47	0.12						
Faculty of graduation	Faculty of education	16	3.95	0.34	−0.03	Student	−0.94	39	0.35	−0.30	[−0.93, 0.33]
	Faculty of arts and sciences	25	4.08	0.45	−0.28						
School location	City center	22	3.93	0.48	−0.07	Student	−1.63	39	0.11	−0.51	[−1.13, 0.12]
	Rural area	19	4.15	0.34	−0.63						

Welch’s *t*-test was used when the assumption of homogeneity of variance was violated. The 95% confidence intervals for Cohen’s *d* were reported for all independent-group comparisons. In the Findings section, however, the confidence intervals reported alongside Welch’s test refer to the raw mean difference between the groups rather than to the standardized effect size.

According to the skewness values presented in Table 5, the TSESOSL scores of the teachers who participated in the study exhibited a normal or approximately normal distribution across the demographic variable groups (skewness coefficient −1 < skewness < +1). Levene’s test indicated that the homogeneity of variance assumption was violated for the gender comparison (*F* = 7.56, *p* = 0.009); therefore, Welch’s *t*-test was used for this comparison. The gender comparison was not statistically significant, Welch’s *t* (37.08) = −1.27, *p* = 0.211, 95% CI [−0.37, 0.08]. The comparisons based on professional experience, type of faculty graduated from, and workplace location were also not statistically significant (*p* > 0.05). Given the limited sensitivity associated with the sample and subgroup sizes, these non-significant results are inconclusive and should not be interpreted as demonstrating equivalence between the demographic groups.

The findings obtained from the analysis of qualitative data on physics teachers’ self-efficacy in organizing educational trips to out-of-school learning environments are presented in Table 6, supported by representative quotations from the teacher interviews.

**Table 6:** Physics teachers’ self-efficacy for organizing educational field trips to out-of-school learning environments according to demographic variables.

Theme	Code	Sub-codes		Illustrative participants							
				Female	Male	≤14 years	≥15 years	Faculty of education	Faculty of arts and sciences	City center	Rural area
Self-efficacy	Self-confidence	Belief in one’s ability to implement	Self-perceived theoretical competence	✓	✓	✓	✓	✓	✓	✓	✓
			Need for prior preparation		✓		✓		✓		✓
			Belief in providing appropriate explanations		✓		✓		✓		✓
			Implementation based on student readiness		✓		✓		✓	✓	
		Openness to development and innovation	Awareness of the need for in-service training	✓		✓		✓			✓
			Addressing deficiencies through inquiry		✓		✓		✓	✓	
		Lack of implementation experience		✓	✓	✓	✓	✓	✓	✓	✓
		Knowledge of materials and equipment	Lack of knowledge	✓	✓	✓	✓	✓	✓	✓	✓
		Knowledge of legal permission procedures	Lack of knowledge	✓	✓		✓		✓	✓	✓
			Partial knowledge	✓	✓	✓	✓	✓	✓		✓
	Moderate self-confidence	Lack of implementation experience	Experiencing apprehension	✓		✓			✓	✓	
					✓			✓			✓
		Belief in one’s ability to implement		✓		✓			✓	✓	
		Openness to development and innovation	Addressing deficiencies through inquiry		✓	✓		✓			✓
		Knowledge of materials and equipment	Lack of knowledge	✓	✓	✓		✓	✓	✓	✓
		Knowledge of legal permission procedures	Having knowledge		✓	✓		✓			✓
	Lack of self-confidence	Lack of implementation experience		✓			✓		✓	✓	
		Belief in learning through inquiry		✓			✓		✓	✓	
		Knowledge of materials and equipment	Lack of knowledge	✓			✓		✓	✓	
		Knowledge of legal permission procedures	Partial knowledge	✓			✓		✓	✓	

A check mark indicates that at least one interviewed teacher in the demographic group expressed the corresponding subcode. The same teacher may be represented under multiple subcodes. Check marks do not indicate participant frequencies or the prevalence of a view within a group.

Several self-efficacy-related views were represented in interview accounts across the demographic categories. It was determined that the teachers described their ability to plan and implement out-of-school learning activities as self-perceived ability to implement, moderate self-confidence, and lack of self-confidence. Within the Belief in One’s Ability to Implement theme, perceived theoretical competence was represented in accounts across the demographic categories. This finding is supported by the statement of P1, “I am theoretically competent; I have a good command of the subject.”, and the statement of P8, “I do not lack knowledge; I know it theoretically.” Within this theme, references to students’ readiness, prior preparation, and the ability to provide appropriate explanations were represented in accounts from male teachers, teachers with 15 years or more of professional experience, graduates of faculties of arts and sciences, and teachers working in rural areas. The statement of P9, a male teacher, “I do not think I will experience any difficulties at the high school level. I can easily answer the students’ questions.” supports the sub-code of Belief in Providing Appropriate Explanations. The need for in-service training was represented in accounts from female teachers, teachers with 14 years or less of professional experience, graduates of faculties of education, and teachers working in rural areas, whereas accounts from the other demographic categories included references to addressing deficiencies through inquiry. Interview accounts within the Belief in One’s Ability to Implement theme also described limited implementation experience and gaps in knowledge of the materials and equipment needed for these activities. Interview accounts within this theme also included reports of limited or partial knowledge of legal permission procedures.

Within the Moderate Self-Confidence theme, limited implementation experience was represented across the demographic categories except among teachers with 15 years or more of professional experience. Apprehension associated with limited experience was represented in accounts from female teachers, teachers with 14 years or less of professional experience, graduates of faculties of arts and sciences, and teachers working in the city center. Despite this apprehension, it was determined that these groups of teachers maintained their belief that they would be able to implement these activities. Among the teachers within the Moderate Self-Confidence theme, male teachers, teachers with 14 years or less of professional experience, graduates of faculties of education, and teachers working in rural areas stated that they could address their deficiencies regarding organizing educational field trips to out-of-school learning environments through inquiry, indicating that they were open to development and innovation. This finding is supported by the statement of P4, a teacher with 14 years or less of professional experience, “If I decide to do it, I will research it and learn. Learning new things is always good.” Interview accounts within the Moderate Self-Confidence theme included reports of limited knowledge of the materials and equipment needed for these activities. Knowledge of legal permission procedures was also represented within this theme. It is noteworthy that teachers with 15 years or more of professional experience were not represented within the Moderate Self-Confidence theme. These patterns describe the distribution of themes within the interview sample and do not indicate demographic differences in self-efficacy.

All subcodes within the Lack of Self-Confidence theme were based on P10’s views. P10 was a female teacher with 15 or more years of professional experience, had graduated from a faculty of arts and sciences, and was working in a city center. Although P10 stated that she had no implementation experience, she indicated that she believed she could address her implementation-related deficiencies through inquiry. She also stated that she lacked knowledge of the materials and equipment required for out-of-school learning activities and had only partial knowledge of the legal permission procedures. P10’s statement regarding implementation experience, “I have not done it.”, and the statement regarding the legal procedures, “I know that permission must be obtained, and that is all. However, I do not know the process completely.” support the relevant codes.

### 3.3 Findings regarding the examination of physics teachers’ anxiety about organizing field trips to out-of-school learning environments in terms of gender, professional experience, type of faculty graduated from, and workplace location

Physics teachers’ anxiety about organizing field trips to out-of-school learning environments was examined in relation to gender, teaching experience, type of faculty from which they graduated, and school location. Accordingly, the findings obtained from the quantitative and qualitative data are presented using a holistic approach and following a systematic sequence.

To determine whether the participating teachers’ scores on the Teacher Anxiety Scale for Organizing Out-of-School Learning (TASOOL) differed according to gender, professional experience, type of faculty graduated from, and workplace location groups, descriptive statistics were first calculated, and the normality of the score distributions was examined. Taking the normality of the dependent variable into consideration, comparisons among the groups of the independent variables were conducted using the appropriate statistical analysis method. The descriptive statistics calculated for the participating teachers’ TASOOL scores according to the groups of the independent variables, together with the results of the analyses, are presented in Table 7.

**Table 7:** Analysis results of the participants’ TASOOL scores according to the groups of the independent variables.

Variable	Group	*N*	*M*	*SD*	Skewness	Test	*t*	*df*	*p*	Cohen’s *d*	95% CI for *d*
Gender	Female	14	2.81	0.27	−0.10	Welch	−0.02	38.37	0.99	−0.00	[−0.28, 0.28]
	Male	27	2.81	0.61	−0.11						
Teaching experience	14 years or less	19	2.82	0.46	−0.86	Student	0.06	39	0.96	0.02	[−0.60, 0.63]
	15 years or more	22	2.81	0.57	0.23						
Faculty of graduation	Faculty of education	16	2.81	0.45	0.59	Student	−0.05	39	0.96	−0.02	[−0.64, 0.61]
	Faculty of arts and sciences	25	2.82	0.56	−0.35						
School location	City center	22	2.84	0.56	−0.01	Student	0.33	39	0.74	0.10	[−0.51, 0.72]
	Rural area	19	2.78	0.47	−0.44						

Welch’s *t*-test was used when the assumption of homogeneity of variance was violated. The 95% confidence intervals for Cohen’s *d* were reported for all independent-group comparisons. In the Findings section, however, the confidence intervals reported alongside Welch’s test refer to the raw mean difference between the groups rather than to the standardized effect size.

According to the skewness values presented in Table 7, the TASOOL scores of the teachers who participated in the study exhibited a normal or approximately normal distribution across the demographic variable groups (skewness coefficient −1 < skewness < +1). Levene’s test indicated that the homogeneity of variance assumption was violated for the gender comparison (*F* = 8.37, *p* = 0.006); therefore, Welch’s *t*-test was used for this comparison. The gender comparison was not statistically significant, Welch’s *t* (38.37) = −0.02, *p* = 0.987, 95% CI [−0.28, 0.28]. The comparisons based on professional experience, type of faculty graduated from, and workplace location were also not statistically significant (*p* > 0.05). Given the limited sensitivity associated with the sample and subgroup sizes, these non-significant results are inconclusive and should not be interpreted as demonstrating equivalence between the demographic groups.

The findings obtained from the analysis of qualitative data on physics teachers’ anxiety about organizing field trips to out-of-school learning environments are presented in Table 8, supported by representative quotations from the teacher interviews.

**Table 8:** Physics teachers’ anxiety about organizing field trips to out-of-school learning environments according to demographic variables.

Theme	Code	Sub-codes		Illustrative participants							
				Female	Male	≤14 years	≥15 years	Faculty of education	Faculty of arts and sciences	City center	Rural area
Anxiety	Concerns about student safety and responsibility	Concerns about student safety and the burden of responsibility		✓	✓	✓	✓	✓	✓	✓	✓
		Difficulty in supervising students	Student level	✓		✓	✓		✓	✓	
			Number of students	✓		✓	✓	✓	✓	✓	✓
	Administrative and legal concerns	Complexity of legal procedures		✓	✓	✓	✓	✓	✓	✓	✓
		Perception that instruction is not taking place	Student	✓		✓		✓		✓	
			School administration	✓	✓	✓	✓	✓	✓	✓	✓
			Parents	✓	✓	✓	✓	✓	✓	✓	✓
	Time and curriculum constraints	Time constraints		✓	✓	✓	✓	✓	✓	✓	✓
		Curriculum intensity		✓	✓	✓	✓	✓	✓	✓	✓
		Impact of the exam-oriented system		✓	✓	✓	✓		✓		✓
	Physical and regional limitations	Regional constraints and lack of suitable environments		✓	✓	✓	✓	✓	✓	✓	✓
		Financial constraints		✓	✓	✓	✓	✓	✓	✓	✓
		Transportation constraints		✓	✓	✓	✓	✓	✓	✓	
	Success anxiety	Concerns about students’ failure to achieve the intended outcomes	Student level	✓	✓	✓	✓		✓	✓	
			Student disinterest and low motivation	✓	✓	✓	✓	✓	✓	✓	✓
		Failure to implement as intended	Concerns due to lack of implementation experience		✓	✓		✓		✓	

A check mark indicates that at least one interviewed teacher in the demographic group expressed the corresponding subcode. The same teacher may be represented under multiple subcodes. Check marks do not indicate participant frequencies or the prevalence of a view within a group.

The five anxiety-related themes were represented across multiple demographic categories in the interview accounts. The teachers’ anxiety regarding these instructional activities was grouped under five main themes: Concerns about Student Safety and Responsibility, Administrative and Legal Concerns, Time and Curriculum Constraints, Physical and Regional Limitations, and Success Anxiety.

Concerns about student safety and increased responsibility were represented in interview accounts across all demographic categories. In addition, the interviews revealed that teachers experienced anxiety regarding the difficulty of supervising students, particularly due to the number of students or their academic level. Concerns about supervising large numbers of students were represented across the demographic categories except in accounts from male teachers. Concerns about supervision related to students’ academic level were represented in accounts from demographic categories other than male teachers, graduates of faculties of education, and teachers working in rural areas. These findings are supported by the statement of P5, a female teacher, “Now all of our classes have more than 30 students. It is difficult to take all of them together. We would also have difficulty supervising them.”, and the statement of P10, “Since they are high school students, we can say that they are easier to supervise. However, because I work at a vocational high school, the students here can be very unruly”.

The complexity of legal procedures was represented in interview accounts across the demographic categories. Concerns that parents or school administrators might perceive these activities as occasions when “no instruction is taking place” were also represented across these categories. Concerns that students might perceive out-of-school learning activities as situations in which “no instruction is taking place” were expressed only by P11.

Time constraints and curriculum intensity were represented as sources of anxiety in interview accounts across all demographic categories. The teachers stated that they might have difficulty allocating sufficient time to out-of-school learning activities due to the intensity of the current curriculum. In addition, views concerning the exam-oriented education system were represented across the demographic categories except in accounts from graduates of faculties of education and teachers working in the city center.

Inadequate regional opportunities, lack of suitable learning environments, and financial constraints were represented as challenges in interview accounts across all demographic categories. Transportation constraints were coded in interview accounts from teachers working in city centers, whereas this code was not identified in the accounts from teachers working in rural areas. These findings are supported by the statement of P6, “There are no places in the city where we live to carry out such activities. For example, there should be an observatory. But there isn’t one.”, and the statement of P3, “I cannot afford the expenses required for the activity. Neither the Ministry of National Education nor the school will cover these costs. Therefore, it is difficult to carry out these activities.” Transportation constraints were also reflected in the statement of P11, a female teacher, “Transportation would be a problem”.

Within the Success Anxiety theme, the teachers stated that they were concerned that they would not be able to implement out-of-school learning activities as intended and that students would not achieve the desired level of success during these instructional activities. Concerns that students might not achieve the desired level of success because of insufficient interest and motivation were represented in interview accounts across all demographic categories. This finding is supported by the statement of P9, “If a student does not fully engage in the activity or if the environment does not attract their interest, they will not learn anything”. Interview accounts also included concerns that differences in students’ academic levels might prevent students from achieving the intended learning outcomes. Concerns about being unable to carry out out-of-school learning activities as planned due to limited implementation experience were expressed only in P2’s interview.

### 3.4 Findings regarding the examination of physics teachers’ self-efficacy and anxiety toward out-of-school learning as predictors of their attitudes toward out-of-school learning

In this study, the relationships among the participating teachers’ self-efficacy in organizing out-of-school learning, anxiety about organizing out-of-school learning, and attitudes toward out-of-school learning were examined. For this purpose, descriptive statistics were calculated for the participants’ attitude, self-efficacy, and anxiety scores related to out-of-school learning, and univariate normality was assessed using skewness coefficients. Descriptive statistics for the scores obtained from the data collection instruments are presented in Table 9.

**Table 9:** Descriptive statistics for the participants’ attitude, self-efficacy, and anxiety scores toward out-of-school learning.

Variable	*M*	*SD*	Skewness
TASOLA score	3.73	0.49	0.60
TSESOSL score	4.03	0.43	−0.14
TASOOL score	2.81	0.51	−0.12

When Table 9 is examined, it is seen that the participants’ scores for all variables exhibited a univariate normal distribution (skewness coefficient −1 < skewness < +1). Multiple linear regression analysis was conducted to examine the relationship between the independent variables of self-efficacy for organizing out-of-school learning and anxiety about organizing out-of-school learning and the dependent variable of attitudes toward out-of-school learning activities. Before conducting the regression analysis, the presence of linear relationships among the variables, each measured on an interval scale, was examined using scatterplots, and the correlation coefficients were calculated. The correlation coefficients among the variables included in the model are presented in Table 10.

**Table 10:** Correlation coefficients among the variables included in the model.

Variable	1	2	3
(1) TASOLA score		0.56^**^	−0.52^**^
(2) TSESOSL score			−0.48^**^
(3) TASOOL score			

^**^Correlation is significant at the 0.01 level.

When the correlation coefficients presented in Table 10 are examined, it is seen that there is a positive linear relationship between the dependent variable, TASOLA score, and the independent variable, TSESOSL score (*r* = 0.56, *p* < 0.01). In contrast, a negative linear relationship was found between the dependent variable, TASOLA score, and the independent variable, TASOOL score (*r* = −0.52, *p* < 0.01). In addition, it was determined that there was no correlation of sufficient magnitude (above 0.70) to indicate a multicollinearity problem among the independent variables (*r* = −0.48). Prior to conducting the regression analysis, the assumptions of linearity between the independent variables and the dependent variable and multivariate normality were assessed using a scatterplot of standardized residuals against standardized predicted values and a normal probability plot (P–P plot) (Tabachnick and Fidell, 2013). The scatterplot showed that the data points tended to cluster around a straight line (the zero line), indicating that the assumption of linearity was satisfied. The normal P–P plot of the standardized residuals indicated that the residuals were approximately normally distributed. Thus, the assumptions required for multiple linear regression analysis were satisfied. A multiple linear regression analysis was conducted to determine the extent to which participants’ TSESOSL and TASOOL scores predicted their TASOLA scores, and the results of the analysis are presented in Table 11.

**Table 11:** Results of the multiple linear regression analysis.

Variable	*B*	*SE*	*β*	*t*	*p*
Constant	2.73	0.913		2.99	0.005
TSESOSL score	0.462	0.164	0.405	2.81	0.008
TASOOL score	−0.306	0.137	−0.322	−2.24	0.031

*R* = 0.63; *R*^2^ = 0.39; adjusted *R*^2^ = 0.36; *F* (2, 38) = 12.28, *p* < 0.01; *N* = 41.

The analysis results presented in Table 11 indicate that the model established to examine the relationship between the two independent variables and the dependent variable is statistically significant, *F* (2, 38) = 12.283, *p* < 0.01. Accordingly, TSESOSL and TASOOL scores were jointly and statistically significantly associated with TASOLA scores. Together, TSESOSL and TASOOL scores accounted for 39% of the variance in TASOLA scores in this sample (*R* = 0.63; *R*^2^ = 0.39; adjusted *R*^2^ = 0.36). When the *t*-test results for the regression coefficients (*B*) of the variables included in the model are examined, it is seen that both the TSESOSL and TASOOL scores are significant predictors of the TASOLA score. According to the standardized regression coefficients (*β*), both TSESOSL and TASOOL were significant predictors of the TASOLA score. Among the two predictors included in the regression model, self-efficacy had a larger standardized regression coefficient than anxiety. According to the results of the multiple linear regression analysis, the regression equation for predicting attitudes toward organizing out-of-school learning is as follows:

$$\begin{array}{c} \text{TASOLA score}=2.729+0.462\;(\text{TSESOSL score})\\ -0.306\;(\text{TASOOL score}) \end{array}$$

In the fitted regression model, a one-unit higher TSESOSL score was associated with a 0.462-unit higher TASOLA score, holding TASOOL score constant. A one-unit higher TASOOL score was associated with a 0.306-unit lower TASOLA score, holding TSESOSL score constant.

## 4 Discussion

### 4.1 Discussion of the findings regarding the examination of physics teachers’ attitudes toward out-of-school learning activities in terms of gender, professional experience, type of faculty graduated from, and workplace location

A statistically significant gender difference was detected in attitudes toward out-of-school learning in this sample, with male teachers reporting higher attitude scores than female teachers. No statistically significant differences were found according to teaching experience, faculty of graduation, or school location. When the interview findings were considered alongside the quantitative gender difference, no systematic contrast was observed between the accounts of female and male teachers that could clearly explain this difference. Interview participants from both gender groups expressed the benefits of out-of-school learning in terms of lasting learning, connections with everyday life, student motivation, and social development. Therefore, the higher attitude scores reported by male teachers in the quantitative analysis could not be explained by a distinct gender-related pattern in the interview data. Furthermore, the fact that the interview sample was limited to eight teachers from each gender group indicates that this qualitative pattern should not be interpreted as evidence of a demographic group difference. Given the small sample and subgroup sizes and the corresponding limited sensitivity of the demographic comparisons, the non-significant findings for teaching experience, faculty of graduation, and school location should be regarded as inconclusive rather than as evidence of no differences between these groups. The statistically significant gender difference should also be interpreted cautiously in light of the small subgroup sizes. The gender difference observed in the present sample differs from the finding of Islahi and Nasrin (2019), who reported no statistically significant gender difference in teachers’ attitudes. The reporting of differing findings regarding professional experience in previous studies (Bilton, 2020; Huang and Shih, 2017) suggests that the relationship between this variable and teachers’ attitudes may vary across different samples and contexts. The absence of a statistically significant difference according to professional experience in the present study should not, however, be interpreted as indicating that professional experience is unrelated to teachers’ attitudes, given the limited sensitivity of the study. From a broader theoretical perspective, Social Cognitive Theory and Self-Determination Theory propose that teachers’ attitudes may be associated not only with individual characteristics but also with self-efficacy beliefs, prior mastery experiences, environmental interactions, and the fulfillment of needs for autonomy, competence, and relatedness throughout their professional careers (Bandura, 1997; Ryan and Deci, 2000). Similarly, the present findings concerning school location and type of faculty graduated from should be considered in light of the study’s limited statistical sensitivity. Previous research indicates that characteristics related to school context and teacher education have been addressed in different ways in relation to teachers’ attitudes. Although it has been suggested that the physical facilities of schools may positively influence attitudes toward out-of-school learning (Mannion et al., 2015), some studies have shown that school location does not create a significant difference (Huang and Shih, 2017). Although no studies were identified that directly examined the relationship between the type of faculty graduated from and attitudes toward out-of-school learning, it is well established that theoretical and practical experiences related to out-of-school learning acquired during teacher education support teachers’ instructional approaches (French et al., 2023). According to the Teacher Cognition framework, teachers’ decisions are shaped not only by their knowledge but also by the combination of their pre-service education, pedagogical beliefs, and previous teaching experiences (Borg, 2003). These theoretical perspectives provide a framework for examining teachers’ attitudes in relation to various individual and contextual factors alongside demographic characteristics. However, the limited sensitivity of the demographic comparisons in the present study does not permit conclusions regarding the relative importance of these factors.

Interview accounts described out-of-school learning as promoting lasting learning, increasing students’ interest and motivation, supporting their social development, and providing opportunities for learning by doing and experiencing. These findings are consistent with those reported by Dönel Akgül and Arabacı (2020), and Fägerstam and Grothérus (2018). From the perspective of Experiential Learning Theory, out-of-school learning environments promote lasting learning by enabling students to interact with the physical environment and construct abstract concepts within the context of everyday life (Beames et al., 2012; Kolb, 2014). From the perspective of Social Cognitive Theory, these environments also contribute to both cognitive and affective development because they support learning through social interaction (Bandura, 1986; Beames et al., 2012). In the interviews, some teachers also described the potential of out-of-school learning to support students’ sense of responsibility, self-confidence, higher-order learning, and career awareness. Previous research has also identified potential benefits of outdoor learning for students’ self-confidence, sense of responsibility, and higher-order learning (Rickinson et al., 2004).

The interviews included contrasting views on the suitability of out-of-school learning for physics instruction. P9 described these activities as suitable because of physics’ connection with nature, whereas P2 considered them generally unsuitable. These accounts illustrate differing individual assessments but do not establish differences by faculty of graduation or school location.

Finally, some interviewees linked their willingness to organize out-of-school learning to students’ interest, while an interview account identified permission procedures as a constraint. This finding is consistent with the results reported by Patchen et al. (2024), and Chrysomalidou et al. (2025). While Self-Determination Theory explains that students’ intrinsic motivation strengthens teachers’ willingness to implement such activities (Ryan and Deci, 2000), Social Cognitive Theory suggests that teachers’ willingness is also associated with environmental conditions and perceived implementation demands (Bandura, 1986; Ertmer and Ottenbreit-Leftwich, 2010). Therefore, it was considered that promoting the wider implementation of out-of-school learning would require systematic measures to reduce bureaucratic procedures and provide implementation support, alongside efforts to foster positive teacher attitudes.

### 4.2 Discussion of the findings regarding the examination of physics teachers’ self-efficacy for organizing educational field trips to out-of-school learning environments in terms of gender, professional experience, type of faculty graduated from, and workplace location

No statistically significant differences in self-efficacy scores were detected according to gender, teaching experience, faculty of graduation, or school location in this sample. The interviews provided further detail about teachers’ perceived capabilities and the conditions affecting their confidence. Given the small sample and subgroup sizes and the limited sensitivity of the comparisons, these non-significant findings should be regarded as inconclusive and do not establish that self-efficacy is equivalent across the demographic groups. Previous studies have reported varying findings regarding the relationship between teacher self-efficacy and demographic characteristics. Varuzzi (2025) reported no statistically significant differences in teacher self-efficacy across demographic variables in the study sample, whereas Klassen and Chiu (2010) noted that findings concerning gender, in particular, have been inconsistent across studies. Although no studies directly examining the type of faculty from which teachers graduated were identified, previous research suggests that the quality of teacher education programs may be important for the development of teacher self-efficacy (Zee and Koomen, 2016). From a theoretical perspective, this relationship can be considered within the framework of Social Cognitive Theory, which proposes that self-efficacy develops through the interaction between individuals, their experiences, and their environment (Bandura, 1997).

The qualitative findings indicated that teachers made different self-evaluations regarding their ability to plan and implement out-of-school learning activities, which were expressed as self-confidence, moderate self-confidence, and lack of self-confidence. Views reflecting a lack of self-confidence emerged from P10’s individual account. Therefore, this pattern was not interpreted as a shared characteristic of female teachers, experienced teachers, graduates of faculties of arts and sciences, or teachers working in city centers. When the quantitative and qualitative findings concerning professional experience are considered together, a notable divergence between the two data sources emerges. In the quantitative analysis, teachers with 15 or more years of professional experience had a numerically higher mean self-efficacy score (*M* = 4.11) than those with 14 years or less of experience (*M* = 3.94), although this difference was not statistically significant. In contrast, in the interview data, the views within the “Lack of Self-Confidence” theme emerged from the individual account of P10, who had 15 or more years of professional experience. This qualitative pattern does not indicate that experienced teachers generally had lower self-efficacy; in particular, the check marks in Table 6 do not represent the frequency of views but indicate that at least one participant within the relevant category expressed the corresponding view. Nevertheless, the different directions suggested by the quantitative and qualitative findings may indicate that overall self-efficacy scale scores and individual accounts of specific implementation experiences reflect different aspects of the same phenomenon. Previous research has also reported differences across professional experience groups in self-efficacy for organizing out-of-school learning activities (Sontay and Karamustafaoğlu, 2017); however, given the small interview sample, the qualitative pattern observed in the present study should not be interpreted as evidence of such a group difference.

Some interviewees expressed confidence in their theoretical knowledge or in answering students’ questions, whereas others described a need for further preparation or training to organize out-of-school learning activities. Accounts describing attention to students’ prior knowledge, advance preparation, and the provision of appropriate explanations also point to the task-specific and pedagogical dimensions of perceived self-efficacy. This finding is consistent with Shulman’s (2013) pedagogical content knowledge framework. Other interview accounts emphasized a need for in-service training related to out-of-school learning. Such requests may reflect awareness of professional development needs. According to the Teacher Cognition framework, teachers’ instructional decisions are shaped by their previous learning experiences, pedagogical beliefs, and evaluations of their own teaching practices (Borg, 2003). In this context, requests for in-service training may reflect recognition of the practical demands of out-of-school learning and openness to professional learning.

One noteworthy finding of the study is that even teachers who expressed confidence identified their lack of implementation experience as a significant limitation. Similarly, teachers with low or moderate levels of self-confidence were found to lack sufficient implementation experience. This finding is consistent with Bandura’s (1997) self-efficacy theory, which identifies mastery experiences as the strongest source of self-efficacy. Some interview accounts linked limited implementation experience with concerns about the risks and responsibilities involved in out-of-school learning. Some of these accounts nevertheless expressed confidence in the ability to prepare for or learn how to implement such activities. According to Bandura (1997), self-efficacy does not require the absence of anxiety; it involves maintaining a belief in one’s ability to perform a task successfully despite uncertainty.

Interviewees described varying levels of knowledge about the materials, equipment, and legal permission procedures involved in out-of-school learning; some reported gaps in their knowledge, while knowledge of legal procedures was also represented in the moderate self-confidence theme. In addition, they stated that they could overcome their deficiencies related to out-of-school learning by conducting research and learning independently. Although the teachers expressed confidence in their general instructional competencies, they evaluated themselves as more limited with respect to specific tasks. This finding is consistent with the views of Tschannen-Moran and Hoy (2001) and Schumann and Sibthorp (2014), who argue that teacher self-efficacy is a task- and context-specific construct.

### 4.3 Discussion of the findings regarding the examination of physics teachers’ anxiety about organizing field trips to out-of-school learning environments in terms of gender, professional experience, type of faculty graduated from, and workplace location

No statistically significant differences in anxiety scores were detected according to gender, teaching experience, faculty of graduation, or school location in this sample. Across the interview groups, teachers described several shared concerns, particularly about safety and the practical demands of organizing out-of-school learning. Given the small sample and subgroup sizes and the limited sensitivity of the comparisons, these non-significant findings should be regarded as inconclusive and do not establish that anxiety levels are equivalent across the demographic groups. When the quantitative and qualitative findings are considered together, the teachers’ overall mean anxiety score (*M* = 2.81) was slightly below the theoretical midpoint of 3.00 on the five-point response scale, whereas concerns related to safety and responsibility, legal procedures, time constraints, and curriculum intensity were represented across all demographic categories in the interview data. These two findings are not necessarily contradictory. While the scale score reflects teachers’ overall level of anxiety, the interviews provide insight into the nature of the sources of anxiety that arise under specific implementation conditions. In addition, the check marks in Table 8 indicate neither the frequency nor the intensity of anxiety; rather, they show that at least one participant within the relevant demographic category expressed the corresponding concern. Therefore, the representation of specific sources of anxiety across different demographic categories in the interviews does not necessarily indicate that teachers had high overall levels of anxiety. The sources of anxiety identified in the interviews also provide a context similar to that reported in previous studies addressing challenges related to safety, time, administrative procedures, and environmental opportunities in the implementation of out-of-school learning (Oberle et al., 2021; Patchen et al., 2024).

The grouping of teachers’ concerns under the themes of Student Safety and Responsibility, Administrative and Legal Concerns, Time and Curriculum Constraints, Physical and Regional Limitations, and Success Anxiety indicates that out-of-school learning is perceived by teachers as a process involving a high level of responsibility. Interview accounts across the demographic categories addressed student safety and the responsibility involved in organizing these activities. Some interviewees also described difficulties in supervising students in relation to class size or students’ academic level. These interview findings can be considered within the broader literature in which challenges associated with the implementation of out-of-school learning, including risk management, preparation, safety, student supervision, time, resources, and lack of school support, have previously been addressed (Oberle et al., 2021; Patchen et al., 2024). From the perspective of Social Cognitive Theory, these concerns are related not only to teachers’ personal levels of anxiety but also to how they evaluate the environmental conditions in which the activity will take place and its possible outcomes (Bandura, 1997; Tschannen-Moran and Hoy, 2001). Therefore, concerns regarding out-of-school learning appear to be associated more with the specific responsibilities and risk evaluations required by these activities than with teachers’ general professional competence.

Interview accounts across the demographic categories addressed legal procedures and concerns about how parents and school administrators might perceive these activities. An account also raised concern about students’ perceptions. According to Borg (2003), teachers’ instructional decisions are influenced not only by their pedagogical knowledge but also by school culture, previous experiences, beliefs, and contextual evaluations. From this perspective, teachers’ concerns regarding the perceptions of parents, students, and school administrators may stem from their cognitive evaluations of how these activities will be interpreted by the school community, even though they believe in the instructional value of out-of-school learning.

Time constraints, curriculum intensity, and the exam-oriented education system were also identified by the teachers as common sources of concern. This finding is consistent with previous research indicating that time constraints, curriculum pressure, and instructional priorities limit the implementation of out-of-school learning activities (Oberle et al., 2021). From the perspective of Cognitive Load Theory, planning out-of-school learning activities requires numerous additional cognitive tasks, including permission procedures, transportation arrangements, safety precautions, preparation of instructional materials, student supervision, and alignment with curriculum objectives (Beames et al., 2012). These additional tasks may increase teachers’ perceived cognitive load, thereby strengthening their anxiety about implementing these activities (Sweller, 1988).

Physical and regional limitations, the lack of suitable out-of-school learning environments, financial constraints, and transportation problems also emerged as contextual factors that teachers associated with their concerns about out-of-school learning. This finding indicates that out-of-school learning cannot be explained solely by teachers’ willingness or pedagogical beliefs, but that environmental opportunities and institutional support mechanisms are also determining factors. In the interviews, transportation constraints were coded in accounts from city-center teachers but not in accounts from rural teachers. Because the table records the presence of a code rather than its frequency, this pattern cannot establish that transportation was a greater concern for one group than the other.

Some interviewees expressed concern that limited implementation experience might prevent them from conducting out-of-school learning activities as intended, while insufficient student interest and motivation might hinder students’ achievement of the intended outcomes. Some interviewees also expressed concern that differences in students’ academic levels could limit students’ achievement of the intended learning outcomes. This finding indicates that teachers’ anxiety is not limited to safety and procedural issues but is also associated with expectations regarding instructional effectiveness. Pekrun (2006) argues that individuals experience higher levels of anxiety when they perceive a low level of control over a task they consider important. This finding is consistent with studies showing that teachers believe the success of out-of-school learning activities is closely associated with students’ prior knowledge, interest, and motivation (Arık, 2022; Utku and Uçak, 2023). Furthermore, the literature emphasizes that teachers’ consideration of students’ characteristics when planning instruction is critical for learning outcomes (Kablan, 2012). In this context, although teachers considered out-of-school learning activities to be valuable, their belief that they would not be able to exercise sufficient control over student motivation, students’ academic levels, implementation experience, and the success of the activities may have increased their success anxiety.

### 4.4 Discussion of the findings regarding the examination of physics teachers’ self-efficacy and anxiety toward out-of-school learning as predictors of their attitudes toward out-of-school learning

In this sample, self-efficacy was a positive statistical predictor of attitude scores, whereas anxiety was a negative statistical predictor; together, they accounted for 39% of the variance in attitude scores (*R*^2^ = 0.39; adjusted *R*^2^ = 0.36). In the regression model fitted to this sample, self-efficacy and anxiety were statistically significant predictors of attitude scores. The demographic characteristics were examined in separate group comparisons and were not included in the same regression model; therefore, their relative predictive contributions cannot be compared with those of self-efficacy and anxiety. The finding that self-efficacy was a strong statistical predictor of attitudes toward out-of-school learning is consistent with Bandura’s Social Cognitive Theory (Bandura, 1997). To the extent that teachers believe they can plan out-of-school learning activities, ensure safety, and effectively guide students, they perceive this instructional approach as more feasible and valuable. In this sample, greater perceived self-efficacy was associated with more positive attitude scores. The cross-sectional findings do not establish that self-efficacy gives rise to those attitudes. Teachers with high levels of self-efficacy have been reported to be more willing to implement innovative instructional approaches, to cope more effectively with challenges encountered during instruction, and to adopt alternative practices that support student learning (Tschannen-Moran and Hoy, 2001; Zee and Koomen, 2016). Furthermore, according to Zee and Koomen (2016), teacher self-efficacy is strongly associated with instructional motivation, instructional quality, student engagement, and openness to instructional innovation. This relationship is also supported by the Teacher Cognition framework (Borg, 2003). Teachers’ instructional decisions are based not only on the knowledge they possess but also on their belief that they can use this knowledge effectively. The qualitative findings further revealed that teachers were confident in their theoretical knowledge of out-of-school learning, were able to take students’ prior knowledge into account, and believed that they could overcome deficiencies in their knowledge through independent inquiry. Taken together, the survey associations and interview accounts suggest that perceived capability and practical preparation are relevant to understanding teachers’ attitudes toward out-of-school learning.

In the current regression model, anxiety toward out-of-school learning was a statistically significant negative predictor of attitude scores; however, this association does not establish a temporal or causal direction between the variables. The interview accounts show that teachers recognized the educational value of out-of-school learning while also describing risks and uncertainties associated with its implementation. These accounts provide context for the negative association between anxiety and attitudes, but they do not establish how that association arises. From an educational psychology perspective, the observed negative association highlights the relevance of anxiety to understanding teachers’ attitudes toward out-of-school learning. According to the Control-Value Theory, individuals experience higher levels of anxiety when they attach high value to a task but perceive that they have insufficient control over its outcomes (Pekrun, 2006). This theoretical framework also suggests that the value teachers attribute to out-of-school learning may be associated with anxiety; therefore, the direction specified in the current regression model should not be regarded as the only theoretically plausible direction. Although out-of-school learning is perceived by teachers as having high educational value, factors such as responsibility for student safety, legal procedures, time management, transportation planning, student supervision, and coping with unexpected situations may reduce their perceived control over the implementation process. Therefore, anxiety may be interpreted not as an indication of individual inadequacy but as a cognitive-affective evaluation associated with teachers’ perceived control over the implementation process. The qualitative findings of the study also support this interpretation. The teachers identified student safety, increased responsibility, legal permission procedures, school administrators’ expectations, time constraints, the exam-oriented education system, and a lack of implementation experience as the primary factors that make out-of-school learning difficult to implement. Similarly, Oberle et al. (2021), and Patchen et al. (2024) reported that safety, organization, time pressure, insufficient financial resources, and limited institutional support negatively influence teachers’ decisions to implement out-of-school learning.

The negative association between anxiety and attitudes highlights the relevance of teachers’ implementation-related concerns. In the interviews, teachers described concerns about student safety, legal procedures, and the practical demands of organizing out-of-school learning. Risk-management guidance, clearer permission procedures, and support from school administrators may be useful areas for future intervention. Whether such measures reduce anxiety, change attitudes, or increase the implementation of out-of-school learning requires further study.

In this sample, the regression model comprising self-efficacy and anxiety accounted for 39% of the variance in attitude scores (*R*^2^ = 0.39; adjusted *R*^2^ = 0.36). This result describes the model’s statistical fit and does not show that these variables caused changes in teachers’ attitudes.

The three scales address related but distinct aspects of out-of-school learning: attitudes toward the activities, perceived ability to organize them, and anxiety about organizing them. Nevertheless, some attitude items refer to the difficulty, preparation, and stress involved in these activities, which are conceptually close to aspects assessed by the self-efficacy and anxiety scales. This overlap, together with the use of self-report measures completed by the same teachers, may have contributed to the observed associations and the variance explained by the regression model. The findings should therefore be interpreted as associations among related measures rather than as evidence that the constructs are entirely independent. Although the scales were administered at different times, their common self-report source may have inflated or otherwise affected the observed correlations and regression coefficients. The present analyses cannot determine how much of these associations reflects substantive relationships and how much, if any, reflects shared measurement method.

The contribution of the present study is to examine self-efficacy, anxiety, and attitudes toward out-of-school learning together in a physics-teacher sample, extending previous research with classroom teachers (Yıldırım and Efendıoglu, 2023) and science teachers (Arık, 2022; Utku and Uçak, 2023). While the out-of-school learning literature has largely focused on identifying structural barriers such as safety, time, cost, and bureaucratic procedures (Oberle et al., 2021; Patchen et al., 2024), the present study considers teachers’ accounts of structural barriers alongside the observed associations of self-efficacy and anxiety with their attitudes. Thus, the study provides empirical evidence that integrates the out-of-school learning literature with the cognitive and affective perspectives of educational psychology.

## 5 Limitations

This study has several limitations. The quantitative sample comprised 41 physics teachers recruited through non-probability sampling from a single province, which limits the generalizability of the findings. The small sample and demographic subgroup sizes also limited the sensitivity of the group comparisons. The sensitivity analysis indicated that the study was adequately powered only for relatively large standardized group differences (approximately *d* = 0.90–0.95). Accordingly, the non-significant demographic comparisons should be regarded as inconclusive rather than as evidence of equivalence between groups, and the significant gender difference in attitude scores should be interpreted cautiously given the small subgroup sizes. In addition, the cross-sectional design supports associations and statistical prediction but does not establish causal relationships. Given the cross-sectional nature of the study, the temporal direction of the relationships among self-efficacy, anxiety, and attitudes cannot be determined; therefore, the ordering of the variables in the current regression model should not be regarded as the only theoretically plausible model. Structural barriers were identified in the interviews but were not included as measured variables in the quantitative regression model. Therefore, the study did not test whether anxiety mediates an association between structural barriers and attitudes. Finally, demographic characteristics and psychological variables were not evaluated together in the same predictive model, so their relative contributions to attitude scores cannot be determined from the present analyses. Potential overlap in item content and the use of self-report scales completed by the same teachers may have contributed to shared-method variance in the observed associations. The scales were administered at different times, but this procedural separation does not rule out shared-method effects.

## 6 Conclusion and implications

In this sample, self-efficacy was positively associated with physics teachers’ attitudes toward out-of-school learning, whereas anxiety was negatively associated with these attitudes. A statistically significant gender difference was found in attitudes toward out-of-school learning, with male teachers reporting higher attitude scores than female teachers. The remaining demographic comparisons of attitudes, self-efficacy, and anxiety were not statistically significant. However, because the sensitivity analysis indicated limited power to detect small-to-moderate group differences, these non-significant results should be regarded as inconclusive rather than as evidence that the demographic groups are equivalent. These findings should be interpreted in light of the small, non-probability sample. Furthermore, they underscore the relevance of self-efficacy and anxiety in understanding teachers’ attitudes toward out-of-school learning.

The interview findings identified practical barriers to out-of-school learning, while the quantitative findings showed associations of self-efficacy and anxiety with teachers’ attitudes. Whether changes in practical conditions or psychological support lead to greater implementation remains to be tested. Support aimed at strengthening teachers’ self-efficacy and addressing their implementation-related concerns may be considered for development and evaluation. In this context, increasing practice-oriented in-service training, providing guidance for planning out-of-school learning activities, and ensuring that school administrators offer institutional support to teachers may contribute to the more effective implementation of these practices.

In this sample, the regression model comprising self-efficacy and anxiety accounted for 39% of the variance in attitude scores (*R*^2^ = 0.39; adjusted *R*^2^ = 0.36). This result does not establish the mechanisms underlying these associations. Future research may examine the effects of variables such as school leadership, institutional support, and implementation experience on these relationships. In addition, studies conducted with larger samples, across different subject areas, and using longitudinal research designs may strengthen the generalizability of the present findings.

## Data Availability

The original contributions presented in the study are included in the article/supplementary material, further inquiries can be directed to the corresponding author.
